# First report of novel mutation (c.790del) on *SQSTM1* gene on a family with childhood onset of progressive cerebellar ataxia with vertical gaze palsy

**DOI:** 10.1002/ccr3.6203

**Published:** 2022-08-09

**Authors:** Hossein Jalali, Atefeh Khoshaeen, Mohammad Reza Mahdavi, Mahan Mahdavi

**Affiliations:** ^1^ Thalassemia Research Center, Hemoglobinopathies Institute Mazandaran University of Medical Sciences Sari Iran; ^2^ Sinayemehr Research Center Mazandaran University Sari Iran; ^3^ Department of Biomedical Engineering, Science and Research Branch Islamic Azad University Tehran Iran

**Keywords:** PCR‐sequencing, progressive cerebellar ataxia, SQSTM1 gene

## Abstract

SQSTM1 gene encodes a protein called p62 that acts as an autophagy receptor in the degradation of protein molecules. A homozygous deletion variant that changes the frame shift in the SQSTM1 gene named c.790 Del A .T was detected in case childhood onset and progressive neurodegeneration with ataxia, and gaze palsy.

## 1 INTRODUCTION

Located on long arm of chromosome 5 (5q35.3), the *SQSTM1* gene encodes a protein called p62 that was first identified by Jaekyoon Shin and his colleagues.^1^ P62, also known as sequestosome‐1, is a scaffold protein, playing a pivotal role in modulating enzyme function through several domain interactions. P62 acts as an autophagy receptor in the degradation of protein molecules. In the past decade, studies have shown that mutations on *SQSTM1* gene are associated with several diseases including Paget's disease of bone (PDB), liver cancer, breast cancer, obesity, diabetes, and neurodegenerative diseases (including Ataxia, Dystonia, And Gaze Palsy, Childhood‐Onset).^2^

With the advent of next‐generation sequencing (NGS), a larger number of the new mutations causing various syndromes were identified. In the presented case, we describe a patient from north of Iran who presented with progressive cerebellar ataxia and vertical gaze palsy due to homozygous *SQSTM1* mutation detected by WES (Whole Exome Sequencing).

## 2 CASE PRESENTATION

A 53‐year‐old female patient with childhood onset and progressive neurodegeneration with ataxia, dystonia, and gaze palsy was referred for genetic counseling and subsequent DNA analysis. The patients had severe scoliosis, short neck, ataxia, oculomotor apraxia, dystonia, dysmetria, dysarthria, cognitive abnormality, Pes Cavus, and Dysdiadochokinesia.

**FIGURE 1:**
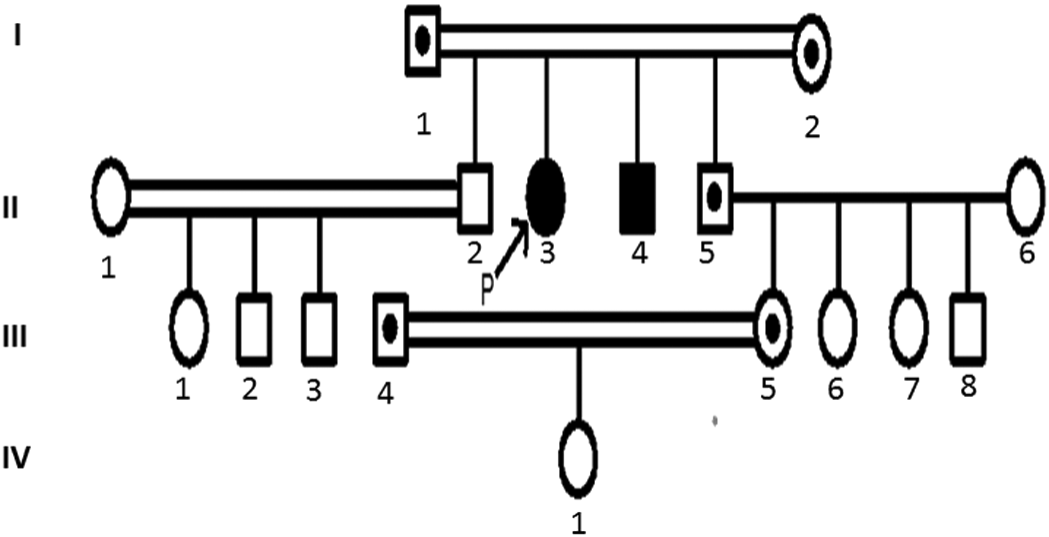
Pedigree chart of the family with SQSTM1 mutation: The case was born of a consanguineous marriage and she had a brother with similar clinical features and one of her nieces was carrier for c.790 del A mutation. III‐4, III‐5: Confirmed Heterozygote, I‐1, I‐2, and II‐5: Suspected Heterozygote, II‐4: Suspected Homozygote.

She was born of a consanguineous marriage, and family history showed that she had a brother with similar clinical manifestations (Figure 1).

A written informed consent was obtained and in order to explore the probable disease‐causing variants, Genomic DNA was isolated from the whole blood sample, and whole‐exome sequencing (WES) was conducted by Illumina platform. A homozygous deletion variant that changes the frame shift in the *SQSTM1* gene named c.790del was detected. Analysis of the identified variant using Mutation Taster tools predicted the variant as a disease‐causing frame‐shift mutation.

**FIGURE 2:**
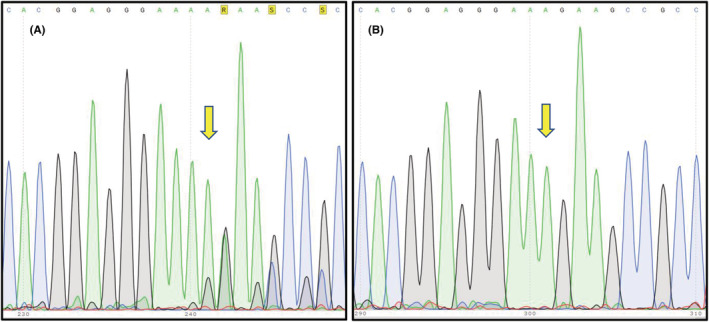
Targeted sequencing result of c.790delA mutation on SQSTM1 gene indicates a homozygote state for affected case (B) and a heterozygote genotype for her niece (A), respectively.

In order to verify the identified variant and explore the mutation among other members of the family, polymerase chain reaction (PCR) amplification and targeted sequencing of the identified variant were applied. Accordingly, the DNA sequence of *SQSTM1* gene was obtained from the NCBI database, and locus‐specific primers (5‐CTGAATTGGAGAAAGAGAAAGG‐3 and 5‐AAGGCGATCTTCCTCATCTG‐3) were designed using the Oligo7 software. Then, the samples were sequenced by Sanger sequencing method via Applied Biosystems 3130xl Genetic Analyzers. Finally, the obtained sequences were assessed by Codon code software. We also detected the mutation in heterozygote state in two nieces of the affected case who got married to each other. Although the carrier couples have a normal girl, the prenatal diagnosis for further pregnancies is recommended (Figure 2).

## 3 DISCUSSION

Neurodegenerative diseases that result from the progressive loss of function and subsequent death of neurons in the central and peripheral nervous systems cause major threats to human health including severe disability, or even early death.^3^ The identification of related genetic variants in both common and rare cases provides crucial new insights into pathophysiology and basic cellular processes of the disease. Almost in all of the neurodegenerative diseases, ubiquitin‐enriched misfolded protein inclusions are main cellular characteristics.^4^ P62 is an adaptor protein for ubiquitinated substrates selected for Macroautophagy.^5^ The absence of p62 was observed in patients with childhood‐onset neurodegenerative disease^6^ and *SQSTM1* knockdown in zebrafish model leads to the neurodegenerative disorders ALS/FTLD in which abnormal motor behavior and shortening of motor neurons were obvious.^7^, ^8^

In 2016, Haack et al. for the first time described childhood and adolescence onset of neurodegenerative syndrome, demonstrating mainly with gait abnormalities and ataxia in four different families. Exome sequencing in nine subjects showed three different biallelic loss of function variants in *SQSTM1* gene^6^: The c.286C>T and c.311_312del variants that affect all three predicted SQSTM1 isoforms and c.2T>A variant that only affects the start codon of one isoform. Muto et al. in 2018 detected 11 affected individuals from three unrelated consanguineous families two of which were from central Iran where the c.934_936delinsTGA and c.875_876insT mutations were identified in the affected cases and another family from Italy with c.301+2T>A splice site mutation (Table 1).^9^ Cerebellar ataxia, dysarthria, cognitive impairment, and gaze palsy were observed among Iranian patients while in the presented case in addition to the mentioned clinical manifestations dystonia and dyskinesia were also detected. Moreover, Iridoplegia, dysautonomic features such as orthostatic hypotension and sudomotor dysfunction, along with other non‐motor symptoms was also reported in patients from Mexico and Jourdan that was not observed in the presented case.

**TABLE 1:** Characteristics of the pathogenic variants on SQSTM1 gene

Pathogenic variant	Origin	Confirmed cases	Age on onset	Location on the gene
c.790del	North of Iran (presented case)	1	Early childhood	Exon 7
c.311_312del	United Arab Emirates	3	10, 10, 10	Exon 3
c.286C>T	Finland, Kurdish Origin	3	7, 8, 8	Exon 2
c.2T>A	Germany	3	10, 12, 14	Exon 1
c.934_936delinsTGA	Central Iran	2	10, 12	Exon 6
c.875_876insT	Central Iran	7	10 to 11	Exon 6
c.301+2T>A	Italy	2	6 and 12	Intron 2
c.712_713insTCCTCCGAGTGTGAATTTCCTGA	India	1	11	Exon 5

Some variants of *SQSTM1* gene are common to both PDB and ALS/FTD, others have been suggested to be ALS/FTD specific,^10^ and in rare cases like the presented case, the mutation was reported linked to childhood‐onset progressive cerebellar ataxia with vertical gaze palsy. The confusing variety of clinical symptoms and inheritance patterns associated with *SQSTM1* variants indicates complex genotype/phenotype relationships and introducing the new variants can leads to the better understanding of the gene function.

## AUTHOR CONTRIBUTIONS

Hossein Jalali performed the test and analyzed the results, and involved in writing of the article. Atefeh Khoshaeen visited the case as a physician. Mohammad Reza Mahdavi involved in writing and editing of the article. Mahan Mahdavi performed the tests.

## FUNDING INFORMATION

None.

## CONFLICT OF INTEREST

The authors have no conflict of interest.

## ETHICAL APPROVAL

Written informed consent was obtained from the patient's brother.

## CONSENT

Written informed consent was obtained from the patient's brother to publish this report in accordance with the journal's patient consent policy.

## Data Availability

All data underlying this article are incorporated into the article.
